# Role of Probiotics in Human Health

**DOI:** 10.7759/cureus.31313

**Published:** 2022-11-09

**Authors:** Harsh Bodke, Sangita Jogdand

**Affiliations:** 1 Pharmacology, Jawaharlal Nehru Medical College, Datta Meghe Institute of Medical Sciences, Wardha, IND

**Keywords:** lactobacilli, immunomodulation, probiotics, intestinal disease, gut microflora

## Abstract

Certain bacteria, known as probiotics, have had a vastly beneficial effect on people's health; considering their benefits they have been mixed with a wide variety of foods for several decades now. The ability of probiotics to modify the immunological response of the host, antagonize pathogenic microbes, or compete for adhesion sites with pathogenic microorganisms is related to the action of probiotics against microorganisms. Infections of the digestive tract, irritable bowel, lactose intolerance, allergies, infections of the urogenital tract, cystic fibrosis, and various cancers can all be prevented and treated with the use of probiotics. They can reduce the side effects of various antibodies. In the field of oral health, dental caries, periodontal disease, and bad breath can be prevented and treated with the use of probiotics. The findings of several of these clinical studies indicate that probiotics may be beneficial in the treatment and prevention of various diseases and health issues. Validation of a significant number of these clinical investigations is necessary before the results can be applied to the clinical setting. Clinical studies play an important part in such investigations, and in the not-too-distant future, the outcomes of such trials will determine whether or not probiotics are effective in the treatment of disease. This article will attempt to provide a summary of the available literature on the benefits that these probiotics have with regard to health and disease.

Probiotics are foods and/or supplements that contain non-pathogenic microbes such as bacteria and yeast that colonize the gut and can potentially yield a variety of health benefits. Research into the various ways in which probiotic bacteria could be used in the treatment of intestinal disorders is ongoing. Thanks to clinical studies and laboratory experiments, we now know more about how probiotics affect gut microbiome disorders. Studies can prove that probiotics can alleviate a variety of gastrointestinal ailments and improve overall health. This article concentrates on probiotics and commensal microbes, as well as their potential role in gut microbiome-related illnesses. In this section, we mark certain areas that need further work and studies so as to enhance our understanding of how probiotics help in the treatment and reduction of chances of gastrointestinal diseases.

## Introduction and background

Human beings consume a significant number of pathogens every day, primarily bacteria. For several decades, probiotic microorganisms have been utilized in several diets due to their positive effects on human health [1,2]. The Agriculture Organization of the United States and the World Health Organization [3] defined probiotics in 2001 as bacteria that, when given to a host at adequate levels, improve their health. Regarding probiotics, Lactobacillus and Bifidobacterium are the two most frequently used genera. These bacteria are generally thought of as harmless due to their ability to survive in the body to cure and prevent diseases, unlike the usual pathogens. Along with that, these microorganisms have played a vital role in the process of fermenting milk and food preservation for many years [4]. Numerous randomly selected clinical trials have shown that probiotic strains are safe and effective in providing users with their benefits. These benefits include the prevention of acute diarrhoea, Crohn's disease, cardiovascular and urogenital infections, cancer, lactose intolerance, cystic fibrosis, dental caries, and oral diseases. Bacteria may also be beneficial in preventing tooth decay, treating periodontal disease, and reducing oral malodor. Probiotics' beneficial role in preventing inflammatory disorders is an ever-expanding list [5]. Over time, scientists have become highly inquisitive about discovering, analyzing, and studying species with probiotic properties. The role of probiotics in health and illness is summarized in this article and is derived from reviewed studies. Human intestinal health can be improved with the use of probiotics. A mixture of prebiotics has also been proposed. Consequently, the probiotics market has grown rapidly, trying to make probiotics a part of food and supplements [6]. The use of probiotics has been recommended to improve immunity and general health. It is now understood that the commensal human gut microbes may contribute to the onset of metabolic illnesses such as obesity, diabetes, and inflammatory bowel disease. Interestingly, it has been seen that the consumption of probiotics significantly increases the prognosis and management of such disorders. A concise explanation of the idea, purpose, and connection between gut microbiome-related disorders and probiotics is given in this review.

## Review

How probiotics work in our body

There are various ways the probiotics can work inside our body: Interacting and stimulating the growth of the body's good commensal microbes, and inhibiting the growth of pathogens inside the human body. They sometimes also increase the host's antigenic response time, which in turn increases the synthesis of antimicrobial compounds and can also block the site where the pathogen might bind. Furthermore, probiotics exhibit adherence and (at least temporary) colonization of the human body, increasing the duration of retention and leading to sustained probiotic function [5-12]. Several probiotics used as food materials in today's world have been listed in the table below (Table 1).

**Table 1:** Probiotics used in various products in the world

Probiotic bacteria	Product	Country
1. Bifidobacterium bifidum	Infant formula	Turkey
2. B. breve	Drink	Japan
3. B. lactis	Infant formula	Israel
4. Lactobacillus acidophilus	Yogurt	UK
5. L. casei Shirota	Drink	Argentina, Australia, Belgium, Brazil, China, Germany, Japan, Indonesia, UK, USA, Taiwan
6. L. casei	Yogurt	France, Colorado/Arizona (USA)
7. L. lactis L1A	Yogurt	Sweden
8. L. plantarum 299v	Fruit drink, Ice cream, Recovery drink, Oat mixture	Sweden

Probiotics and their benefits

**Figure 1:**
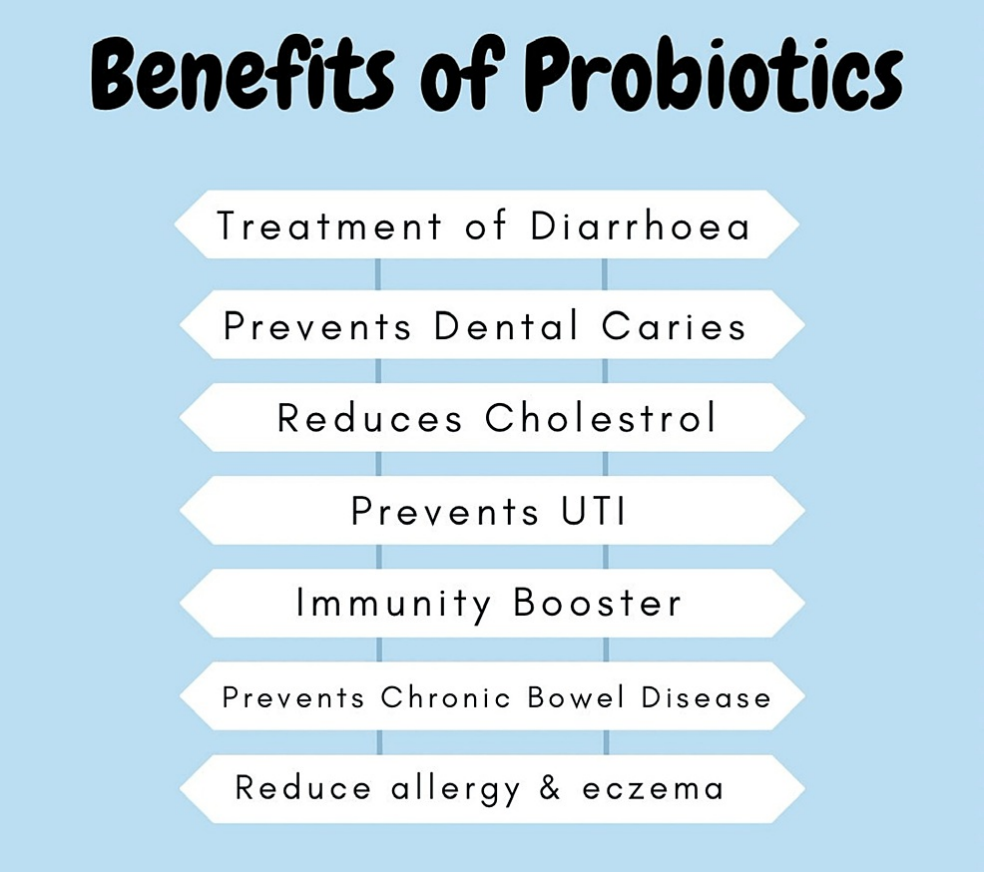
Benefits of Probiotics This illustration has been created by the author of this study. UTI: Urinary tract infection

Figure 1 lists a few benefits of probiotics for a healthy human life. These include a large variety of uses not just for a particular part but almost everywhere in our body. In the gastrointestinal tract, lies their well-known benefit of improving our digestion and reducing the amount of cholesterol. Their other uses involve the treatment of diarrhea and the prevention of inflammatory bowel diseases. Furthermore, the benefits will include preventing dental caries and strengthening our immune system, especially during allergic conditions. The constant growth of harmful microorganisms can also be inhibited by probiotics to an extent. Bifidobacterium creates glutamine, which preserves the integrity of the mucosa and improves the mucosal barrier's defenses [13-14]. Many studies have proved that probiotics help deal with different kinds of diarrhea, such as travelers' diarrhea, antibiotic-induced diarrhea, and rotavirus-related diarrhea in small children [15,16]. Irritable bowel syndrome has a poorly understood cause, and hence treatment for such people is challenging. However, introduction to the Enterococcus strain PR88 as an oral probiotic showed clinical improvement in patients. Research has persisted for the use of probiotics and led to some intriguing results of probiotics and their utilization in complex conditions [17].

The positive effects of probiotics on patients with food allergies are a result of their combined impact on the gut's non-immunologic and immunologic defense barriers. The natural dietary protein's immunomodulatory characteristics are altered by lactobacilli. Probiotics affect the immune system by stimulating the gastrointestinal lymphoid tissue's lymphoid cells [18-21]. Probiotics' effect on atopic dermatitis has been studied in depth. The intensity of eczema and the number of different processes in the given population decreased with the consumption of living and heat-neutralized probiotic bacteria [22]. Lactobacilli protects women from developing urinary tract infections (UTIs) [23]. Using the distal urethral isolates L. rhamnosus GR-1 and L. fermentum B-54 with RC-14 as a substitute for anti-gram-positive bacilli activities and hydrogen peroxide production, studies have shown that the two-strain combination works. For a year, researchers found that using single or multiple two capsules a week vaginally can help prevent the recurrence of UTIs [23-26]. Human probiotics reduce cholesterol. Probiotics cause direct digestion of lipids, which influences cholesterol production. No research showed statistically significant changes in cholesterol levels [27].

Dental caries, a multi-factor bacterial illness, is characterized by acid decalcification of the outer layer of our teeth. To be effective in preventing dental caries, a probiotic must be able to attach itself to teeth coatings and mix into the microbial flora that composes the dental biofilm. To halt the spread of cariogenic bacteria, it must also go to fight and repel them. Research has found that the cariogenic species Streptococcus sobrinus could not grow on a hydroxyapatite surface because only Streptococcus thermophilus and Lactobacillus lactis ssp. lactis could form a biofilm there. The ability of W. cibaria isolates to inhibit S. mutans' capacity to create microbes both in vitro and in vivo, and furthermore to prevent this microbe from proliferating, has been demonstrated recently [28-30]. Periodontal diseases are classified into two categories: gingivitis and periodontitis. Gum disease, unlike periodontitis, is a short-lived, non-debilitating inflammation of the gums that affects only the unattached gingiva and not the alveolar bone [31]. Most efforts to prevent periodontal disease and make sure a full recovery focus on getting rid of pathogens and strengthening the outer boundary, which also makes a person less likely to get sick. Periodontal health may be improved if these good bacteria find a way to establish themselves in dental biofilm and inhibit harmful bacterial growth and metabolism then and there. A wide range of data analysis has found probiotic bacteria to be safe. Few clinical studies have been published on how these probiotics could be beneficial in the treatment of periodontal disease [32,33]. Laboratory research is therefore the key source of data on probiotics that deliberately address periodontal tissues. Gum or lozenges containing probiotics improved the periodontal health of patients with periodontal disease. Many people have foetor ex ore or bad breath, which is commonly referred to as halitosis. Volatile sulfur compounds (VSCs) are primarily produced by Gram-negative anaerobes in dental plaque and on the surface of the tongue and are the main reason for bad breath in the oral cavity. This ailment has also been shown to respond well to bacterial therapy. Probiotic strains of bacteria from healthy people's native oral microbiota could be used to start replacing the bacteria allegedly involved in halitosis as an adjunct for prevention and treatment [34].

How probiotics affect oral health

The oral effects of probiotics are unknown. There is a slight reduction in gum disease with the use of probiotics. Substances made by lactic acid bacteria cause inflammation. Most studies show that probiotics can eliminate infections by outcompeting bacteria for bonding surfaces and nutrients [35,36]. None of the reviewed research read by the author indicated the harmful effects of bacteriotherapy [37-43]. Taipale et al. reported that two probiotic intervention group patients and two placebo control group patients were eliminated due to gastrointestinal discomfort and atopic eczema, respectively. Bacteriotherapy has proven to be helpful in strengthening the immune system in people with HIV and cancer. This review focuses on periodontal disease and dental caries (each of which has been described separately).

Caries and probiotics

Caries has several causes. Pathogenesis includes the host's immune system, microbiota (mainly S. mutans), and diet. Microbiological reasons are the predominant cause of tooth caries, though all of these factors must be present. Only 19 of 725 articles on PUBMED met the inclusion criteria for this review. Only two studies (systematic review) looked explored the effects of probiotic use on dental caries. The authors' research was hampered by having to compare the colony-forming unit (CFU) counts of S. mutans and Lactobacilli earlier and now when they use probiotics. S. mutans CFU levels decreased significantly in-between bacteriotherapy, but not Lactobacilli. S. mutans CFU levels in the intervention group were lower (105 CFU/ml) and less elevated (>106 CFU/ml) than in the comparison group or when Lactobacilli CFU counts were compared. Study design, treatment duration, probiotic strains and concentrations, and follow-up period have varying methodologies in the published studies.

Probiotics role in human gut-associated microbiome diseases

Human Gut Microbiota Dysbiosis

Microbial dysbiosis is a term used to describe an unbalance in the structure and function of intestinal microorganisms [44]. Antibiotic use, bacterial infections, and changes in the diet all contribute to the problem, which has become more prevalent in the modern era. Irritable bowel syndrome (IBS), celiac disease, and other intestinal illnesses are associated with a lack of useful bacteria in the gut. Beneficial probiotics in the gastrointestinal tract inhibit pathogenic microbes from trying to infiltrate and grow by competing for space and resources [45]. Re-establishing healthy commensal microbes and trying to prevent infections in patients following antibiotic therapy is among the most vital uses of probiotics. They are usually used for curing antibiotic-associated diarrhea (AAD), which occurs whenever the microbial community is disrupted. Carbapenem-resistant Clostridioides difficile (previously Clostridium difficile), a disease-causing bacterium, is one of the major causes of AAD. Previous reviews and meta-analyses have shown that probiotics when used in conjunction with other treatments, can help avoid AAD in patients of any age group [46]. Probiotics help stop diarrhea induced by C. difficile both in adults and children [47,48]. There was no perceivable increased likelihood of side effects in this study's findings that probiotic diagnosis may reduce AAD incidence by 51%. Furthermore, a study on this topic has shown that Lactobacillus rhamnosus and Saccharomyces boulardii are seen to be highly effective in protecting AAD.

Chronic Bowel Disease (Inflammatory Bowel Disease)

Inflammatory bowel disease (IBD) is a chronic inflammatory condition that affects the digestive system. IBD includes Crohn's disease (CD), ulcerative colitis (UC), and indeterminate colitis (IC), which are distinguishable based on where the GI tract inflammation is located [49]. IBD is assumed to be caused by an aberrant immune system response; while the exact etiology is still unclear, stress and an unbalanced diet are claimed to be the potential causes. According to some studies, IBD pathogenesis may account for the presence of gut pathogens. Furthermore, numerous investigations have demonstrated that the microbiome of IBD patients differs from that of healthy individuals. Additionally, it has been proposed that preserving the balance of the gut microbes may be crucial for preventing IBD [50]. Probiotics have drawn a lot of attention recently as a potential treatment to alter the microbe's positive effects on IBD. Probiotics, for instance, have been utilized to treat ulcerative colitis and induce remission [51-52]. However, a recent study found that probiotic supplementation appears to be a workable adjuvant therapy for UC and not for CD in individuals with inflammatory bowel disease. As a reason, there is presently insufficient knowledge concerning probiotics' efficacy to make broad recommendations for treatment in CD patients.

Crohn's disease (CD), an irritable bowel disorder that involves losing weight, constipation, temperature, lethargy, and abdominal pain, affects the entire gastrointestinal tract (GIT). The onset of CD is influenced by several factors, including genetic, environmental, and microbial [52], although the exact cause is still unknown. CD's symptoms can be treated with various medications, however, the disease has no known cure. Lower immune system function and intestinal inflammation can be treated with immunosuppressants and steroid medications [53]. Similarly, probiotics may offer an additional technique to standard therapy. It has been proved that early therapy was preferable to postoperative probiotic feeding in CD patients. A more recent study, however, found that performing adjuvant multi-strain probiotic treatment had no appreciable impact on inflammatory responses in CD patients. Additional study is required to clarify the function of probiotics due to discrepancies in the findings of clinical trials employing probiotic supplements to treat CD.

Sources of probiotics

**Figure 2:**
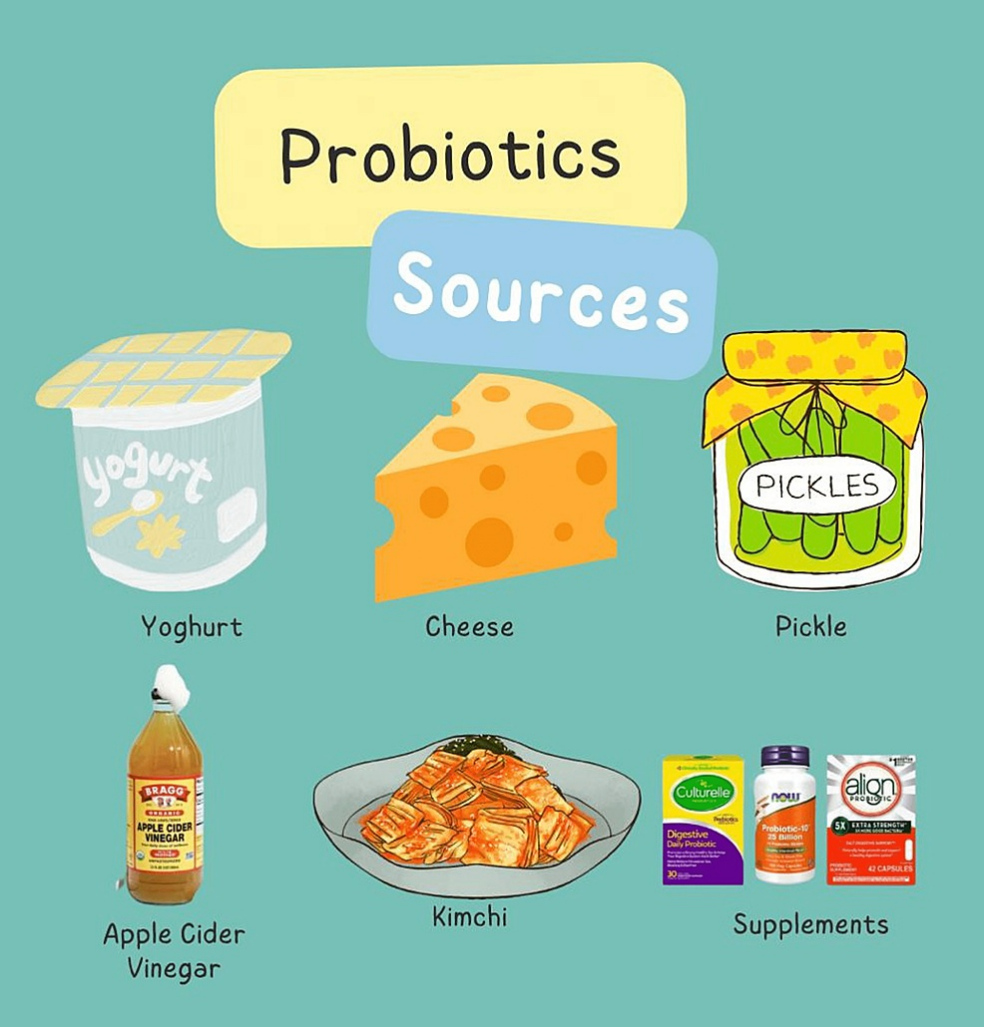
Sources of Probiotics This illustration has been created by the author of this study.

Live bacteria and yeasts are known as probiotics, and they may be good for one's health. They can be found in some meals and supplements as well as in the human digestive system. Probiotic bacteria are beneficial. They are found all over the body, although most people only think of the stomach and intestines when they think of them. Fermented foods such as yogurts and kimchi are sources of probiotics. Additionally, probiotic supplements are also available (Figure 2).

## Conclusions

The study of the intricate connections between diet and health, as it relates to probiotics, is a relatively recent area of medical research. Although preliminary evidence from multiple research labs has been encouraging. This research will make it possible to identify the probiotics that are most suitable for a given application and the optimum delivery systems, such as food items (cheese, milk, and yogurt) or supplements (chewing gum, lozenges). Because these bacteria have the benefit of being completely adapted to the human oral ecology, the presence of probiotics in the native human microflora should be studied. Previous studies regarding the benefits of probiotics on the human intestinal microbiome have shown to have promising findings for the cure of gut-related disorders. Additionally, in recent years there has been an increase in the number of studies that investigate how microorganisms contribute to patients' intestinal illnesses. Although much more research that is hypothesis-driven is required, there is a possibility that probiotics could be utilized as therapeutic options or prevention measures for diseases that are related to the individual gut flora.

The rapid advancement of next-generation sequencing (NGS) technologies has made it possible to gain a deeper comprehension of the roles that specific probiotics play in the protection and enhancement of human health. It is quite likely that these probiotics are related to the equilibrium of the bacteria in the intestines, as well as the regulatory control of intestinal disorders. These various omics technologies, such as microbial genome sequencing, transcriptomics, metagenomics, and sometimes even metabolomics, have increased our knowledge of the isolation and recruitment of suitable probiotic strains, the confirmation of their genuine probiotic effect as well as the mechanisms involved, and the evaluation of an individual's health effects in in vitro and in vivo experiments. This has led to a greater depth of knowledge regarding these aspects of probiotic research. Therefore, to build innovative probiotic strains and maybe pharmabiotic treatments, up-to-date information on bacteria and their effects on humans at an omics level, which has only recently begun towards becoming available, is required. The incorrect use of certain microbes, such as L. rhamnosus, has indeed been linked to the development of septicemia, septic shock, or endocarditis in individuals who exhibit symptoms of inflammation of the digestive organs. For this reason, it is essential, while treating particular patients, to make use of the appropriate quantity of probiotics.
